# Authors' Reply

**DOI:** 10.18502/jovr.v17i4.12344

**Published:** 2022-11-29

**Authors:** Natasha F S Cruz, Katia S Santos, Mateus L Matuoka, Niro Kasahara

**Affiliations:** ^1^Department of Ophthalmology, Irmandade da Santa Casa de Misericordia de Sao Paulo, Sao Paulo, Brazil; ^2^Santa Casa de Sao Paulo School of Medical Sciences, Sao Paulo, Brazil.; ^4^Natasha Cruz: https://orcid.org/0000-0002-5209-9204; ^5^Niro Kasahara: https://orcid.org/0000-0003-4101-0304

## Dear Editor,

We appreciate D. Fleischman's comments on the recent article by Cruz et al.^[1]^ We deeply respect the colleague's opinion on the subject.

The first mention of an algorithm for the estimation of the cerebrospinal fluid pressure (CSFP) appeared in the prospective observational comparative study by Xie et al.^[2]^ The authors included 72 neurology patients who underwent CSFP measurement by lumbar puncture and 3.0-Tesla orbital magnetic resonance imaging for different clinical reasons. After adjusting for body mass index (BMI) and mean arterial blood pressure (MABP), the authors developed three algorithms for the associations between CSFP and orbital subarachnoid space width as follows: (a) CSF-P = 9.31 × OSASW (at 3 mm) + 0.48 × BMI + 0.14 × MABP – 19.94; (b) CSF-P = 16.95 × OSASW (at 9 mm) + 0.39 × BMI + 0.14 × MABP – 20.90; and (c) CSF-P = 17.54 × OSASW (at 15 mm) + 0.47 × BMI + 0.13 × MABP – 21.52. Later, using the data obtained in Xie et al´s study, Jonas and colleagues from the same study group calculated a formula to estimate the CSFP of a normal population.

Performing a multivariate analysis in the training group with the lumbar CSFP measurements as dependent variable and age, BMI and blood pressure as independent variables revealed that estimated CSFP was best described by the formula of Estimated CSFP [mmHg] = 0.44 × BMI × [kg/m ] + 0.16 × Diastolic Blood Pressure [mmHg] – 0.18 × Age [Years] - 1.91.^[3,4]^ In fact, Xie was the coauthor of this second paper and since the data for the first study was used to estimate the CSFP, we thought that paper should be cited and receive credit.

This formula has been validated in the Brazilian population. Kasahara et al compared the real CSFP and the estimated one by the algorithm in a small cohort of 39 patients scheduled for lumbar puncture for different medical reasons.^[5]^Using the Bland-Altman plot of the differences between the two techniques against their averages, most data points were positioned between the two limits of agreement indicating concordance between the two methods. The authors highlighted that the use of the equation was not for clinical grounds; however, it might be a suitable surrogate method to predict CSFP in clinical studies.^[5]^

The following quote is attributed to Sir Issac Newton: “If I have seen further it is by standing on the shoulders of Giants.”^[6]^ Far from considering ourselves as giants, we believe that Dr Cruz's paper is a small contribution to the emerging large body of evidence of the role of CSFP in the pathogeny of primary open-angle glaucoma. This paper might inspire other researches to further elucidate the complexity of CSFP and glaucoma. In science, researchers have to use whatever is available to develop their research and, in many instances, the methods are not ideal. In this study specifically, the measurement of CSFP by lumbar puncture for study purposes only – with no clear medical reason – would definitely be unethical. Other methods for noninvasive measurement of CSFP have been developed and although some techniques may show great potential for specific applications, none of these methods can fully replace an invasive technique by lumbar puncture and none has yet been considered the gold standard for clinical science.^[7]^ In the meantime, we believe that surrogate methods, particularly the proxy algorithm, are valid instruments to estimate CSFP in clinical studies and no study using such methods should be disregarded.
